# Supplementary material for: Increased Alveolar Heparan Sulphate and Reduced Pulmonary Surfactant Amount and Function in the Mucopolysaccharidosis IIIA Mouse
Source: Cells. 2021 Apr 8;10(4):849. doi: 10.3390/cells10040849 (PMC8070179; doi:10.3390/cells10040849)
Supplement: Supplementary file 1 [file cells-10-00849-s001.pdf]

Supplementary Material:

Table S1: Diagnostic MS/MS scans for head groups

b = second injection, c = third injection and d = fourth injection

MS	Scan	MS/MS Mode	Lipids	Range (m/z)	
**ES+**	b	Precursor of 184	PC	400:900	PC species containing the phosphorylcholine head group
**ES+**	c	Neutral Loss of 141	PE	400:900	PL species containing the phosphorylethanol-amine head group
**ES-**	c	Precursor of 153	PG, PA, PS & PI	400:900	Acidic PL species generating the glycerophosphate fragment ion (phospatidylglycerol, phosphatidic acid, phosphatidylserine and phosphatidylinositol head group)
**ES-**	c	Precursor of 241	PI	400:900	PI species containing the phosphoinositol head group
**ES-**	c	Neutral Loss of 87	PS	400:900	PS species containing the serine head group
**ES+**	c	Precursor of 264	Ceramide	480-680	Species containing the ceramide headgroup
**ES+**	c	Precursor of 369	Cholesteryl esters	500-750	Any cholesteryl esters
**ES+**	c	Neutral Loss of 35	Diacylglycerol	350-700	Any diacylglycerol species
**ES-**	d	Precursor of 227	14:0	580-900	Fatty acid 14:0
**ES-**	d	Precursor of 241		580-900	Not relevant to our study
**ES-**	d	Precursor of 253	16:1	580-900	Fatty acid 16:1
**ES-**	d	Precursor of 255	16:0	580-900	Fatty acid 16:0
**ES-**	d	Precursor of 271		580-900	Not relevant to our study
**ES-**	d	Precursor of 277	18:3	580-900	Fatty acid 18:3
**ES-**	d	Precursor of 279	18:2	580-900	Fatty acid 18:2
**ES-**	d	Precursor of 281	18:1	580-900	Fatty acid 18:1
**ES-**	d	Precursor of 283	18:0	580-900	Fatty acid 18:0
**ES-**	d	Precursor of 299	20:6	580-900	Fatty acid 20:6
**ES-**	d	Precursor of 301	20:5	580-900	Fatty acid 20:5
**ES-**	d	Precursor of 303	20:4	580-900	Fatty acid 20:4
**ES-**	d	Precursor of 305	20:3	580-900	Fatty acid 20:3
**ES-**	d	Precursor of 327	22:6	580-900	Fatty acid 22:6
**ES-**	d	Precursor of 329	22:5	580-900	Fatty acid 22:5
**ES-**	d	Precursor of 331	22:4	580-900	Fatty acid 22:4

Table S2: PC species values

Molecular species Generic formula	Structure	Mass
**PC30:0**	PC16:0/14:0	706
**PC32:1**	PC16:0/16:1	732
**PC32:0**	PC16:0/16:0	734
**PC34:2**	PC16:0/18:2	758
**PC34:1**	PC16:0/18:1	760
**PC34:0**	PC18:0/16:0	762
**PC36:4**	PC16:0/20:4	782
**PC36:3**	PC18:1/18:2	784
**PC36:2**	PC18:0/18:2	786
**PC36:1**	PC18:0/18:1	788
**PC38:6**	PC16:0/22:6	806
**PC38:5**	PC18:1/20:4	808
**PC38:4**	PC18:0/20:4	810
**PC40:7**	PC18:1/22:6	832
**PC40:6**	PC18:0/22:6	834

Table S3: Complete data for wildtype, heterozygous, control and MPS IIIA groups

		Species	Wildtype	n	Heterozygous		Control	MPS IIIA	*n*	*p*-value
**Heparan sulphate**										
Tissue	ng/mg protein		64.85 ± 9.0	9	75.13 ± 25.5	8	70.00 ±19.21	3511 ± 584.5	11	<0.0001*
BALF	ng/mL		14.78 ± 4.0	9	18.86 ± 8.2	7	16.56± 6.25	400.2 ± 98.3	13	<0.0001*
**Gangliosides**										
Tissue	pmol/mg protein	GM3 d18.1/16.0	7.66 ± 4.21	9	6.70 ± 2.85	8	7.21 ± 2.46	12.26 ± 2.53	11	<0.0001*
Tissue	pmol/mg protein	GM3 d18.0/22.0	16.85 ± 4.2	8	15.49 ± 2.13	8	16.17 ± 3.3	22.33 ± 7.57	11	0.0077*
Tissue	pmol/mg protein	GM3 d18:1/24:1	28.34 ± 11.49	9	17.99 ± 4.467	8	23.47 ± 2.46	27.69 ± 2.76	11	0.2746
Tissue	pmol/mg protein	GM3 d18:1/24:0	7.93 ± 3.24	9	6.43 ± 2.88	8	7.23 ± 0.75	9.28 ± 1.10	11	0.1201
Tissue	pmol/mg protein	GM1 d18:1/16:0	12.54 ± 5.16	9	10.88 ± 3.11	8	11.76 ± 1.04	13.18 ± 1.08	11	0.3689
**BMP**										
Tissue	pmol/mg protein	Total	78.86 ± 16.23	10	81.98 ± 13.96	8	80.25± 14.91	106.66 ± 17.03	11	0.0002*
Tissue	pmol/mg protein	18.1/18.1	11.75 ± 2.96	10	12.47 ± 2.75	8	12.05± 2.81	22.10 ± 3.91	10	<0.0001*
Tissue	pmol/mg protein	18.1/18.2	5.73 ± 1.42	10	6.20 ± 1.28	8	5.94± 1.34	9.63 ± 1.69	10	<0.0001*
Tissue	pmol/mg protein	18.1/20.4	2.90 ± 0.59	10	2.93 ± 0.81	8	3.18 ±1.00	3.99 ± 0.73	10	0.0038*

Tissue	pmol/mg protein	18.1/22.4	0.46 ± 0.18	10	0.51 ± 0.16	8	0.48 ± 0.17	1.03 ± 0.20	10	<0.0001*
Tissue	pmol/mg protein	18.1/22.5	1.88 ± 0.60	10	1.90 ± 0.22	8	1.89 ± 0.51	3.47 ± 0.64	10	<0.0001*
Tissue	pmol/mg protein	18.1/22.6	6.68 ± 1.63	10	7.09 ± 1.68	8	6.87 ± 1.62	10.63 ± 2.32	10	<0.0001*
Tissue	pmol/mg protein	20.4/22.6	0.95 ± 0.22	9	1.08 ± 0.32	8	1.01 ± 0.27	1.33 ± 0.32	10	0.0106*
Tissue	pmol/mg protein	22.5/22.6	1.13 ± 0.38	10	1.10 ± 0.24	8	1.12 ± 0.32	1.72 ± 0.51	10	0.0006*
Tissue	pmol/mg protein	22.6/22.6	5.44 ± 1.72	10	5.72 ± 1.37	8	5.57 ± 1.53	7.80 ± 2.48	10	0.0064*
Tissue	pmol/mg protein	16:0/16:0	11.81 ± 2.50	10	12.03 ± 1.62	8	11.91 ± 0.493	12.05 ± 0.362	10	0.8423
Tissue	pmol/mg protein	16:1/16:0	2.04 ± 0.55	10	2.23 ± 0.32	8	2.13 ± 0.108	2.16 ± 0.095	10	0.8347
Tissue	pmol/mg protein	16:1/18:1	1.59 ± 0.44	10	1.65 ± 0.33	8	1.61 ± 0.092	1.88 ± 0.100	10	0.0757
Tissue	pmol/mg protein	18:0/16:0	1.03 ± 0.20	10	1.04 ± 0.18	8	1.04 ± 0.044	1.08 ± 0.042	10	0.5165
Tissue	pmol/mg protein	18:1/16:0	20.36 ± 3.87	10	20.74 ± 2.64	8	20.53 ± 0.776	22.46 ± 0.883	10	0.1301
Tissue	pmol/mg protein	18:1/18:0	4.30 ± 0.97	10	4.46 ± 0.87	8	4.37 ± 0.213	4.746 ± 0.209	10	0.2609
Tissue	pmol/mg protein	20:4/20:4	0.49 ± 0.27	10	0.51 ± 0.24	8	0.50 ± 0.058	0.51 ± 0.039	10	0.8823
BALF	pmol/mL	Total	757.4 ± 111.5	9	672.0 ± 68.52	7	720 ± 102.1	685.0 ± 119.0	13	0.4012
BALF	pmol/mL	18.1/18.1	95.73 ± 17.38	9	83.09 ± 11.39	7	90.2 ± 15.96	77.07 ± 17.28	13	0.0430*
BALF	pmol/mL	18.1/20.4	19.04 ± 3.64	9	14.19 ± 6.20	7	16.92 ± 5.35	12.76 ± 4.17	13	0.0300*
BALF	pmol/mL	18.1/22.6	45.13 ± 6.70	9	30.84 ± 13.05	7	38.88 ± 12.07	29.8 ± 8.92	13	0.0325*
BALF	pmol/mL	16:0/16:0	145.7 ± 20.33	9	136.0 ± 21.85	7	141.50 ± 5.221	147.80 ± 6.352	13	0.4429
BALF	pmol/mL	16:1/16:0	21.72 ± 3.57	9	20.44 ± 3.95	7	21.16 ± 0.917	20.31 ± 0.866	13	0.5116
BALF	pmol/mL	16:1/18:1	12.61 ± 2.50	9	11.62 ± 1.61	7	12.18 ± 0.536	10.67 ± 0.486	13	0.0510
BALF	pmol/mL	18:0/16:0	17.15 ± 2.37	9	16.85 ± 4.26	7	17.02 ± 0.802	17.03 ± 0.971	13	0.9937
BALF	pmol/mL	18:0/18:0	1.79 ± 0.34	9	1.71 ± 0.23	7	1.76 ± 0.072	1.81 ± 0.106	13	0.6299
BALF	pmol/mL	18:1/16:0	246.2 ± 44.62	9	227.4 ± 25.89	7	238.0 ± 9.43	235.9 ± 10.09	13	0.8803
BALF	pmol/mL	18:1/18:0	73.71 ± 12.81	9	66.86 ± 7.08	7	70.72 ± 2.737	66.94 ± 3.841	13	0.4195
BALF	pmol/mL	18:1/18:2	42.91 ± 4.67	9	38.22 ± 6.14	7	40.86 ± 1.425	38.21 ± 2.764	13	0.3751
BALF	pmol/mL	18:1/22:4	1.78 ± 0.40	9	1.426 ± 0.61	7	1.63 ± 0.129	1.31 ± 0.116	13	0.0839
BALF	pmol/mL	18:1/22:5	10.09 ± 2.31	9	6.74 ± 2.49	7	8.62 ± 0.719	6.84 ± 0.526	13	0.0648
BALF	pmol/mL	20:4/20:4	1.00 ± 0.37	9	0.8586 ± 0.56	7	0.92 ± 0.113	0.79 ± 0.110	13	0.4180
BALF	pmol/mL	20:4/22:6	3.36 ± 0.62	9	2.54 ± 1.58	7	3.00 ± 0.294	2.53 ± 0.270	13	0.2601
BALF	pmol/mL	22:5/22:6	3.03 ± 0.54	9	1.81 ± 1.16	7	2.50 ± 0.260	2.24 ± 0.220	13	0.4667
BALF	pmol/mL	22:6/22:6	16.46 ± 2.15	9	11.35 ± 6.48	7	14.22 ± 1.277	13.03 ± 1.484	13	0.5449
**Cholesterol**										
Tissue	pmol/mL	Total	2422 ± 839.9	10	2601 ± 556.1	8	2501 168.2	2721 154.7	10	0.3490
Tissue	pmol/mL	Free	913.0 ± 519.1	10	991.3 ± 263.3	8	947.8 ± 415.7	977.7 ± 220.0	10	0.8350
Tissue	pmol/mL	CE18:3	23.76 ± 12.38	10	23.26 ± 5.16	8	23.54 ± 9.60	25.74 ± 8.159	10	0.5455
Tissue	pmol/mL	CE18:2	491.4 ± 280	10	512.6 ± 106.4	8	500.8 ± 215.1	504.7 133.3	10	0.9592

Tissue	pmol/mL	CE20:4	302.3 ± 175.5	10	348.7 ± 141.4	8	322.9 ± 158.5	338.7 ± 71.29	10	0.7688
Tissue	pmol/mL	CE20:3	27.67 ± 13.15	10	31.66 ± 10.57	8	29.44 ± 11.90	30.64 ± 8.95	10	0.7845
Tissue	pmol/mL	CE22:6	68.01 ± 42.93	10	75.09 ± 27.83	8	71.16 ± 36.16	77.97 ± 14.81	10	0.5761
BALF	pmol/mL	Total	5647 ± 758.3	9	5386 ± 982.7	7	5533 ± 843.2	4416 ± 968.2	13	0.0100*
BALF	pmol/mL	Free	4283 ± 584.6	9	4149 ± 597.3	7	4224 ± 574.2	3858 948.6	13	0.2105
BALF	pmol/mL	CE18.3	27.92 ± 6.62	9	26.25 ± 12.55	7	29.00 ± 6.09	12.5 ± 5.18	13	0.0002*
BALF	pmol/mL	CE18.2	660.8 ± 187.7	9	624.3 ± 270.3	7	644.8 ± 219.9	290.4 ± 117.4	13	0.0001*
BALF	pmol/mL	CE20.4	561.1 ± 218.8	9	498.5 ± 269.5	7	533.7 ± 235.8	210.3 ± 87.06	13	0.0004*
BALF	pmol/mL	CE22.6	114.7 ± 37.1	9	87.82 ± 45.95	7	102.90 ± 42.05	44.59 ± 19	13	0.0001*
**Phospholipid composition and alterations in total alveolar phospholipid pool size**										
BALF	%PL	PC	82.93 ± 0.25	3	81.54 ± 1.66	6	82.73 ± 0.42	80.29 ± 4.25	13	0.1515
BALF	%PL	PG	15.29 ± 0.37	3	16.36 ± 1.79	6	15.25 ± 0.33	17.54 ± 4.13	14	0.1659
BALF	%PL	PE	1.27 ± 0.10	3	1.43 ± 0.11	6	1.38 ± 0.13	1.12 ± 0.28	13	0.0004*
BALF	%PL	PS	0.21 ± 0.02	3	0.35 ± 0.15	6	0.305 ± 0.13	0.230 ± 0.08	14	0.1060
BALF	%PL	SM	0.21 ± 0.08	3	0.23 ± 0.07	6	0.226 ± 0.07	0.254 ± 0.11	14	0.5002
Tissue	%PC	16:0/14:0	3.60 ± 0.45	3	3.86 ± 0.43	7	3.78 ± 0.43	3.56 ± 0.22	16	0.0905
Tissue	%PC	16:0/16:1	12.83 ± 1.23	3	12.57 ± 1.00	7	12.64 ± 1.00	12.79 ± 0.62	16	0.6434
Tissue	%PC	16:0/16:0	28.93 ± 1.00	3	28.43 ± 0.90	7	28.58 ± 0.90	28.40 ± 1.22	16	0.6937
Tissue	%PC	16:0/18:2	8.01 ± 0.73	3	8.98 ± 0.25	7	8.69 ± 0.61	8.96 ± 0.78	16	0.3540
Tissue	%PC	16:0/18:1	14.88 ± 0.39	3	15.01 ± 0.70	7	14.97 ± 0.60	14.38 ± 1.09	16	0.1313
Tissue	%PC	18:0/16:0	1.77 ± 0.12	3	1.61 ± 0.26	7	1.66 ± 0.23	1.73 ± 0.22	16	0.4086
Tissue	%PC	16:0/20:4	7.28 ± 0.87	3	7.09 ± 0.48	7	7.15 ± 0.58	7.53 ± 0.64	16	0.1327
Tissue	%PC	18:1/18:2	2.06 ± 0.18	3	2.34 ± 0.20	7	2.26 ± 0.23	2.32 ± 0.22	16	0.5610
Tissue	%PC	18:0/18:2	4.55 ± 0.44	3	4.64 ± 0.24	7	4.61 ± 0.29	4.66 ± 0.35	16	0.6988
Tissue	%PC	18:0/18:1	3.20 ± 0.11	3	3.36 ± 0.21	7	3.31 ± 0.20	3.22 ± 0.25	16	0.3212
Tissue	%PC	16:0/22:6	3.87 ± 0.60	3	3.62 ± 0.35	7	3.69 ± 0.42	3.60 ± 0.25	16	0.4561
Tissue	%PC	18:1/20:4	1.80 ± 0.11	3	1.85 ± 0.19	7	1.83 ± 0.16	1.75 ± 0.18	16	0.2229
Tissue	%PC	18:0/20:4	5.11 ± 0.27	3	4.80 ± 0.69	7	4.90 ± 0.59	5.22 ± 0.38	16	0.0976
Tissue	%PC	22:5/18:2	0.41 ± 0.05	3	0.43 ± 0.06	7	0.43 ± 0.05	0.40 ± 0.04	16	0.1166
Tissue	%PC	18:0/22:6	1.70 ± 0.28	3	1.42 ± 0.19	7	1.51 ± 0.24	1.49 ± 0.19	16	0.8376
BALF	%PC	16:0/14:0	6.40 ± 0.19	3	6.86 ± 0.85	7	6.72 ± 0.73	6.40 ± 0.55	14	0.2257
BALF	%PC	16:0/16:1	28.96 ± 1.11	3	27.81 ± 1.45	7	28.16 ± 1.40	27.26 ± 1.35	14	0.1282
BALF	%PC	16:0/16:0	43.77 ± 0.61	3	45.07 ± 1.99	7	44.68 ± 1.76	47.08 ± 1.71	14	0.0029*
BALF	%PC	16:0/18:2	5.52 ± 0.57	3	5.60 ± 0.83	7	5.58 ± 0.73	5.19 ± 0.62	14	0.1813
BALF	%PC	16:0/18:1	9.05 ± 1.42	3	9.14 ± 0.53	7	9.12 ± 0.80	8.80 ± 0.73	15	0.3238
BALF	%PC	18:0/16:0	1.04 ± 0.17	3	0.92 ± 0.15	7	0.96 ± 0.15	0.95 ± 0.20	15	0.9610
BALF	%PC	16:0/20:4	1.62 ± 0.08	3	1.35 ± 0.31	7	1.43 ± 0.29	1.32 ± 0.19	14	0.2743
BALF	%PC	18:1/18:2	0.25 ± 0.03	3	0.29 ± 0.07	7	0.28 ± 0.06	0.21 ± 0.06	15	0.0121*
BALF	%PC	18:0/18:2	0.42 ± 0.03	3	0.44 ± 0.10	7	0.41 ± 0.04	0.38 ± 0.06	14	0.1451
BALF	%PC	18:0/18:1	0.24 ± 0.04	3	0.25 ± 0.07	7	0.25 ± 0.06	0.25 ± 0.05	14	0.9101
BALF	%PC	16:0/22:6	1.93 ± 0.35	3	1.42 ± 0.29	7	1.70 ± 0.51	1.48 ± 0.22	14	0.1487
BALF	%PC	18:1/20:4	0.22 ± 0.01	3	0.22 ± 0.08	7	0.22 ± 0.06	0.19 ± 0.03	14	0.2114
BALF	%PC	18:0/20:4	0.30 ± 0.05	3	0.24 ± 0.05	7	0.26 ± 0.05	0.21 ± 0.07	15	0.1059
BALF	%PC	22:5/18:2	0.03 ± 0.01	3	0.03 ± 0.01	7	0.03 ± 0.01	0.02 ± 0.01	15	0.0724
BALF	%PC	18:0/22:6	0.25 ± 0.06	3	0.17 ± 0.04	7	0.19 ± 0.06	0.15 ± 0.05	15	0.1190
BALF	nmol	Total pool size	259.9 ± 64.3	3	271.4 ± 100.4	7	267.6 ± 85.8	134.6 ± 36.7	14	< 0.0001*
**mRNA**										
Tissue	MNE	SP-A	8.06 ± 1.87	15	9.08 ± 2.91	8	8.41 ± 2.28	6.83 ± 1.38	18	0.0184*

Tissue	MNE	SP-B	5.94 ± 1.26	15	6.22 ± 1.59	8	6.04 ± 1.35	5.84 ± 0.78	18	0.5782
Tissue	MNE	SP-C	4.14 ± 1.69	15	4.28 ± 2.10	8	4.19 ± 1.80	2.16 ± 0.84	18	0.0001*
Tissue	MNE	SP-D	0.29 ± 0.05	14	0.31 ± 0.09	8	0.30 ± 0.06	0.24 ± 0.06	19	0.0173*
**Surfactant Protein**										
Tissue	%total protein	SP-A	0.027 ± 0.008	13	0.030 ± 0.007	9	0.028 ± 0.008	0.026 ± 0.010	17	0.3176
Tissue	%total protein	SP-B	0.005 ± 0.002	12	0.006 ± 0.002	8	0.0058 ± 0.002	0.0063 ± 0.002	16	0.3480
Tissue	%total protein	SP-C	0.050 ± 0.036	12	0.050 ± 0.025	9	0.050 ± 0.031	0.040 ± 0.025	18	0.2773
Tissue	%total protein	SP-D	0.0044 ± 0.003	12	0.0072 ± 0.004	10	0.0057 ± 0.004	0.0036 ± 0.003	19	0.0443*
BALF	%total protein	SP-A	0.43 ± 0.11	5	0.39 ± 0.13	4	0.41 ± 0.11	0.25 ± 0.10	13	0.0027*
BALF	%total protein	SP-B	0.034 ± 0.012	6	0.036 ± 0.013	4	0.035 ± 0.012	0.030 ± 0.013	13	0.3393
BALF	%total protein	SP-C	0.022 ± 0.006	6	0.023 ± 0.018	6	0.022 ± 0.012	0.010 ± 0.005	13	0.0024*
BALF	%total protein	SP-D	0.172 ± 0.050	6	0.220 ± 0.129	5	0.194 ± 0.093	0.132 ± 0.046	13	0.0438*
**Captive Bubble Surfactometry - BALF**										
**Initial adsorption –** two way-ANOVA										
BALF	Timepoint	0s				10	64.06 ± 1.25	64.05 ± 3.00	6	
BALF	Timepoint	1s				10	21.09 ± 0.73	29.73 ± 9.78	6	
BALF	Timepoint	5s				10	20.34 ± 0.75	26.94 ± 8.81	6	
BALF	Timepoint	20s				10	19.93 ± 0.55	24.77 ± 8.17	6	
BALF	Timepoint	60s				10	19.63 ± 0.55	22.93 ± 7.27	6	
BALF	Timepoint	180s				10	19.34 ± 0.45	21.68 ± 5.43	6	
BALF	Timepoint	300s				10	19.16 ± 0.46	20.79 ± 4.12	6	
BALF		Time*Genotype interaction								<0.0001*
BALF		Time								<0.0001*
BALF		Genotype								0.0593
**Post expansion adsorption** - two-way ANOVA										
BALF	Timepoint	0s				10	26.47 ± 3.67	36.41 ± 11.25		
BALF	Timepoint	1s				10	23.83 ± 2.44	32.52 ± 10.31		
BALF	Timepoint	5s				10	21.37 ± 0.45	28.42 ± 9.14		
BALF	Timepoint	20s				10	20.80 ± 0.42	24.92 ± 6.28		
BALF	Timepoint	60s				10	20.54 ± 0.37	22.72 ± 4.07		
BALF	Timepoint	180s				10	20.32 ± 0.33	21.03 ± 1.42		
BALF	Timepoint	300s				10	20.07 ± 0.40	20.57 ± 1.20		
BALF		Time*Genotype interaction								<0.0001*
BALF		Time								<0.0001*
BALF		Genotype								0.0253*
**Surface tension**										
BALF	γ(mN/m)	γ_{min}	1.60 ± 0.34	6	1.33 ± 0.27	4	1.49 ± 0.34	8.59 ± 4.83	6	0.0003*
BALF	γ(mN/m)	γ_{max}	28.93 ± 0.05	6	29.57 ± 1.27	4	29.19 ± 0.88	36.77 ± 8.38	6	0.0115*
**Surface area**										
BALF		1st cycle	0.319 ± 0.05	6	0.251 ± 0.025	4	0.291 ± 0.05	0.440 ± 0.07	6	0.0003*
BALF		10th cycle	0.123 ± 0.06	6	0.100 ± 0.02	4	0.114 ± 0.05	0.382 ± 0.14	6	<0.0001*

BALF	20th cycle	0.104 ± 0.04	6	0.092 ± 0.01	4	0.100 ± 0.03	0.379 ± 0.14	6	<0.0001*
**Compressibility**									
BALF	1st cycle	45.15 ± 13.88	6	60.65 ± 10.04	4	51.35 ± 14.31	18.18 ± 15.36	6	0.0006*
BALF	10th cycle	218.6 ± 66.08	6	253.1 ± 54.40	4	232.4 ± 61.08	69.78 ± 57.40	6	0.0001*
BALF	20th cycle	250.9 ± 44.94	6	261.5 ± 36.69	4	255.1 ± 40.01	86.18 ± 67.59	6	<0.0001*
**Hysteresis**									
BALF	10th cycle	0.690 ± 0.723	6	0.898 ± 0.78	4	0.773 ± 0.71	3.04 ± 1.61	6	0.0015*
BALF	20th cycle	0.322 ± 0.367	6	1.165 ± 1.691	4	0.659 ± 1.03	2.73 ± 1.55	6	0.0076*

Data presented as mean ± SD; * *p*-value of ≤0.05 is considered significant between the Control and MPS IIIA groups; BALF – bronchoalveolar lavage fluid; BMP - Bis(monoacylglycerol)phosphate; PC – Phosphatidylcholine, PG – Phosphatidylglycerol, PE – Phosphatidylethanolamine, PS – Phosphatidylserine, SM – Sphingomyelin.
